# Determinants of COVID-19 Vaccine Uptake in Adolescents 12–17 Years Old: Examining Pediatric Vaccine Hesitancy Among Racially Diverse Parents in the United States

**DOI:** 10.3389/fpubh.2022.844310

**Published:** 2022-03-22

**Authors:** Aaliyah Gray, Celia B. Fisher

**Affiliations:** ^1^Department of Psychology, Fordham University, Bronx, NY, United States; ^2^Center for Ethics Education, Fordham University, Bronx, NY, United States

**Keywords:** COVID-19, vaccine hesitancy, pediatric vaccine uptake, racial diversity, adolescents, child, parents, health disparities

## Abstract

As of December 8, 2021, 9.9 million U.S. adolescents ages 12–17 years old remain unvaccinated against COVID-19 (SARS-CoV-2) despite FDA emergency approval of the Pfizer-BioNTech COVID-19 vaccine for use among this age group in May 2021. A slow-down in adolescent vaccine uptake and increased likelihood of hospitalization among unvaccinated youth highlight the importance of understanding parental hesitancy in vaccinating their adolescent children against COVID-19. Racial/ethnic disparities in pediatric COVID-19 infection and hospitalization further underscore the need to examine parental vaccine acceptance and hesitancy among diverse U.S. parent populations. In October 2021, 242 Hispanic and non-Hispanic Asian, Black, and White parents of adolescents ages 12–17 years participated in a national online survey assessing determinants of COVID-19 pediatric vaccine hesitancy. Compared to Asian, Black, and Hispanic parents, non-Hispanic White parents reported reduced odds of having vaccinated their adolescent. Bivariate analyses and a multivariable binomial logistic regression indicated that identification as non-Hispanic White, parental COVID-19 vaccine status and safety measures, COVID-19 misconceptions, general vaccine mistrust and COVID-19 related collectivist and individualist attitudes accounted for 45.5% of the variance in the vaccine status of their adolescent children. Our findings draw attention to the urgent need to consider the COVID-19 beliefs, attitudes, and behaviors of parents from diverse racial/ethnic groups in developing population tailored public health messaging to increase adolescent COVID-19 vaccine uptake.

## Introduction

On May 10, 2021, the United States (U.S.) Food and Drug Administration (FDA) authorized the Pfizer-BioNTech COVID-19 vaccine for emergency use among 12–17 years old (1). This authorization followed ~1.5 million COVID-19 cases among adolescents between March 1, 2020 and April 30, 2021 (1). Although severe disease and hospitalization occurs less often among pediatric populations (2–4), hospitalization rates are approximately 10 times higher among unvaccinated adolescents compared to their fully vaccinated age counterparts (3). As of December 8, 2021, however, just 51% (~12.8 million) of 12–17 year-olds were fully vaccinated (5). With 9.9 million youth remaining unvaccinated, a slow-down in vaccine uptake has become a growing concern (5–7). Consequently, understanding factors contributing to COVID-19 vaccine uptake among this age group is urgent.

The success of vaccination programs for adolescents is dependent on overcoming parental vaccine hesitancy. Studies evaluating COVID-19 vaccine hesitancy and acceptance among samples of adults and parents have reported lower parental income and educational level (6, 8–13), parental concerns about pediatric vaccine safety (6, 8–11, 13–16), and lack of COVID-19 knowledge and related misconceptions among adults (17–20) are associated with vaccine hesitancy. By contrast, COVID-19 vaccine uptake among parents and associated health behaviors (e.g., masking, social distancing, etc.) have been associated with pediatric COVID-19 vaccine acceptance (6, 17, 21, 22). Although collectivist attitudes (emphasizing the needs of the group over the individual) have been found to increase intentions to vaccinate among international parent populations (16, 23), recent data from the Kaiser Family Foundation suggest that parents in the U.S. may emphasize personal choice over collective responsibility in their COVID-19 vaccine attitudes (6).

In the U.S., racial minority children have born the greatest burden of pediatric COVID-19 infection and hospitalization (4, 24–26). To date, however, few studies have examined potential racial/ethnic group differences in the relationship between adolescent COVID-19 vaccine uptake and related parental behaviors and attitudes. Early data suggest there may be higher levels of vaccine hesitancy among Hispanic and non-Hispanic Black parents (4, 9–13). Since a return to pre-pandemic normality is only achievable with high vaccination rates (1, 27), suboptimal vaccination among 12–17 year-olds underscores the importance of identifying determinants of parental pediatric COVID-19 vaccine acceptance and hesitancy for the development of effective public health initiatives. The aims of the current brief report are to (1) examine the extent to which parental demographic factors and COVID-19 behaviors, beliefs, and attitudes jointly and independently account for pediatric COVID-19 vaccine uptake among their 12–17 years old children, and (2) identify similarities and differences in vaccine uptake and the salience of these factors for parent populations of different racial/ethnic backgrounds.

## Methods

Data were collected as a part of a larger online national non-probability survey examining individual and social determinants of parental vaccine hesitancy for pediatric COVID-19 vaccination of children and adolescents in the U.S. Of the 400 English speaking self-identified female guardians (≥21 years old) included in the larger study, a total of 242 Hispanic (*n* = 71, 29.3%) and non-Hispanic Asian (*n* = 48, 19.8%), Black (*n* = 63, 25%), and White (*n* = 60, 24.8%) female guardians reported the vaccine status of a child between the ages of 12–17 years old. Among this current sample, 29.8% did not attend college, 40.1% reported < $20,000 in household income, 24.8% were financially insecure endorsing the item “I cannot make ends meet,” and 64% lived in the Midwest and South. Recruitment was conducted through Qualtrics XM with data collected in October 2021. The research protocol was approved by the university institutional review board.

The primary outcome measure in the current study was the proportion of parents reporting they have vaccinated or have not vaccinated their 12–17 year-old child. The survey adapted items from prior scales to assess the following factors: [1] parental COVID-19 vaccine status and safety measures (e.g., wearing a mask in public, staying away from large crowds and social distancing when meeting people, frequent hand washing, avoiding close-contact spaces and activities) (28); [2] COVID-19 misconceptions (e.g., children have natural immunity and cannot transmit the virus, COVID-19 health risks have been exaggerated, COVID-19 is not any worse than the flu) (28, 29); [3] general vaccine mistrust (e.g., children receive too many vaccines, immunizing children is harmful and this fact is covered up, vaccine effectiveness research data is often fabricated) (30, 31), [4] COVID-19 collectivist attitudes (e.g., getting my child vaccinated for COVID-19 supports the community by stopping the spread of the disease among other children and adults) (32); and [5] COVID-19 individualist attitudes (e.g., getting my child vaccinated for COVID-19 would violate my family's rights) (32). All items were scored on 6-point Likert-type scales with the exception of the four true/false items assessing COVID-19 misconceptions. Demographic information included parents' age, education, household income, financial security, region of residence, employer requirements for vaccination, vaccination status for other household members, and COVID-19 infection among family members.

Descriptive statistics for all variables are provided in Tables 1, 2. Unadjusted binomial logistic regressions were performed to assess differences in determinants of adolescent vaccination status (Table 1) and Pearson Chi-square tests were performed to assess differences among racial/ethnic groups (Table 2) for each demographic variable and the above-mentioned COVID-19 beliefs and attitudes scales. A multivariable binomial logistic regression was performed to evaluate the independent influence of demographic characteristics and COVID-19 beliefs, attitudes, and behavior items and scale scores on adolescent vaccination status. According to G^*^Power *post-hoc* analyses, our sample size achieved sufficient power to assess dichotomous racial difference where non-Hispanic White parents were compared to Hispanic and Non-Hispanic Asian and Black parents (1–β = 0.92) as well as differences between the four racial groups (1–β = 0.91).

**Table 1:** Frequencies/percentages and means/standard deviations for parental demographic characteristics and COVID-19 related beliefs and attitudes and unadjusted bivariate analyses predicting adolescent vaccine status for the full sample.

	**Total sample** ***N* = 242**	**Not vaccinated** ***N* = 180**	**Vaccinated** ***N* = 62**	***P*-value**	**OR (95% CI)**
	***N* (%)**	***N* (%)**	***N* (%)**		
**Parent age**, ***M*** **(*****SD*****)**	35.67 (7.74)	34.66 (7.01)	38.61 (8.99)	0.001^*^	1.07 (1.03, 1.11)
**Race**					
Non-Hispanic Asian	48 (19.8%)	34 (18.9%)	14 (22.6%)	0.53	1.25 (0.62, 2.53)
Non-Hispanic Black	63 (26%)	46 (25.6%)	17 (27.4%)	0.77	1.10 (0.57, 2.11)
Hispanic	71 (29.3%)	48 (26.7%)	23 (37.1%)	0.12	1.62 (0.88, 2.99)
Non-Hispanic White	60 (24.8%)	52 (28.9%)	8 (12.9%)	0.02^*^	0.37 (0.16, 0.82)
**Education**				0.89	1.05 (0.56, 1.98)
Did not attend college	72 (29.8%)	54 (30%)	18 (29%)		
Some college or higher	170 (70.2%)	126 (70%)	22 (71%)		
**Annual household income**				0.46	0.86 (0.58, 1.28)
< $20,000	97 (40.1%)	75 (41.7%)	22 (35.5%)		
Between $20,000 and 50,999	111 (45.9%)	79 (43.9%)	32 (51.6%)		
Between $51,000 and 79,999	22 (9.1%)	19 (10.6%)	3 (4.3%)		
Preferred not to answer	12 (5%)	7 (3.9%)	5 (8.1%)		
**Financial security**				0.83	0.93 (0.48, 1.80)
Cannot make ends meet	60 (24.8%)	44 (24.4%)	15 (25.8%)		
Have just enough or comfortable	182 (75.2%)	136 (75.6%)	46 (74.2%)		
**Region of residence**					
Northeast	34 (14%)	26 (14.4%)	8 (12.9%)	0.76	0.88 (0.38, 2.06)
Midwest	86 (35.5%)	64 (35.6%)	22 (35.5%)	0.99	1.00 (0.55, 1.82)
South	70 (28.9%)	53 (29.4%)	17 (27.4%)	0.76	0.91 (0.48, 1.72)
West	52 (21.5%)	37 (20.6%)	15 (24.2%)	0.55	1.23 (0.62, 2.45)
**Parent vaccine status**				<0.001^*^	14.24 (6.13, 33.11)
No	123 (50.8%)	116 (64.4%)	7 (11.3%)		
Yes	119 (49.2%)	64 (35.6%)	55 (88.7%)		
**Parent's employer requires vaccination^a^**				0.04^*^	2.06 (1.02, 4.17)
No	194 (8.2%)	150 (83.3%)	44 (71%)		
Yes	42 (17.4%)	26 (14.4%)	166 (25.8%)		
I don't know	6 (2.5%)	4 (2.2%)	2 (3.2%)		
**Other adults in their household are vaccinated^a^**				<0.001^*^	8.81 (4.19, 17.69)
No	136 (52.1%)	115 (63.9%)	11 (17.7%)		
Yes	114 (47.1%)	63 (35%)	51 (82.3%)		
I don't know	2 (0.8%)	2 (1.1%)	0%		
**Family members in their household ever had COVID-19**				—	—
No	188 (77.7%)	138 (76.7%)	50 (80.6%)		
Yes	0%	0%	0%		
I don't know	54 (22.3%)	42 (23.3%)	12 (19.4%)		
COVID-19 misconceptions, *M* (*SD*)	0.95 (1.16)	1.11 (1.18)	0.53 (.97)	0.001^*^	0.58 (0.42, 0.81)
Parent COVID-19 safety measures, *M* (*SD*)	4.37 (1.86)	4.21 (1.91)	2.82 (1.61)	0.03^*^	1.21 (1.02, 1.44)
General vaccine mistrust, *M* (*SD*)	3.32 (1.26)	3.58 (1.20)	2.58 (1.15)	<0.001^*^	0.50 (0.38, 0.65)
COVID-19 collectivist attitudes, *M* (*SD*)	4.08 (1.52)	3.75 (1.54)	5.05 (.97)	<0.001^*^	2.12 (1.61, 2.78)
COVID-19 individualist attitudes, *M* (*SD*)	3.24 (1.78)	3.46 (1.71)	2.60 (1.82)	0.001^*^	0.75 (0.63, 0.89)

*Statistical tests: Unadjusted binomial logistic regressions*.

a *“No” and “I don't know” combined in Chi-square analyses*.

* *Indicates significance, p < 0.05*.

**Table 2:** Frequencies/percentages and means/standard deviations for racial/ethnic group differences in adolescent vaccination status and parent characteristics.

	**Total sample** **(*N* = 242)**	**Hispanic and non-Hispanic** **Asian and Black parents** **(*N =* 182)**	**Non-Hispanic** **White parents** **(*N* = 60)**	***P*-value**
	***N* (%)**	***N* (%)**	***N* (%)**	
**Vaccination status of adolescent (ages 12–17)**				0.01^*^
No	180 (74.4%)	128 (70.3%)	52 (86.7%)	
Yes	62 (25.6%)	54 (29.7%)	8 (13.3%)	
**Parent age**, ***M*** **(*****SD*****)**	35.67 (7.74)	35.34 (7.12)	36.95 (9.32)	0.14
**Education**
Did not attend college	72 (29.8%)	55 (30.2%)	17 (28.3%)	0.78
Some college or higher	170 (70.2%)	127 (69.3%)	43 (71.7%)	
**Annual household income**				0.29
Less than $20,000	97 (40.1%)	69 (37.9%)	28 (46.7%)	
Between $20,000 and 50,999	111 (45.9%)	87 (47.8%)	24 (40%)	
Between $51,000 and 79,999	22 (9.1%)	15 (8.2%)	7 (11.7%)	
Preferred not to answer	12 (5%)	11 (6%)	1 (1.7%)	
**Financial security**				0.97
Cannot make ends meet	60 (24.8%)	45 (24.7%)	15 (25%)	
Have just enough or comfortable	182 (75.2%)	137 (74.3%)	45 (75%)	
**Region of residence**				0.13
Northeast	34 (14%)	20 (11%)	14 (23.3%)	
Midwest	86 (35.5%)	67 (36.8%)	19 (31.7%)	
South	70 (28.9%)	54 (29.7%)	16 (26.7%)	
West	52 (21.5%)	41 (22.5%)	11 (18.3%)	
**Parent vaccine status**				0.46
No	123 (50.8%)	90 (49.5%)	33 (55%)	
Yes	119 (49.2%)	92 (50.5%)	27 (45%)	
**Parent's employer requires vaccination^a^**				0.08
No	194 (80.2%)	140 (76.9%)	54 (90%)	
Yes	42 (17.4%)	36 (19.8%)	6 (10%)	
I don't know	6 (2.5%)	6 (3.3%)	0%	
**Other adults in their household are vaccinated^a^**				0.71
No	136 (52.1%)	93 (51.1%)	33 (55%)	
Yes	114 (47.1%)	87 (47.8%)	27 (45%)	
I don't know	2 (.8%)	2 (1.1%)	0%	
**Family members in their household ever had COVID-19**				–
No	188 (77.7%)	149 (81.8%)	39 (65%)	
Yes	0%	0%	0%	
I don't know	54 (22.3%)	33 (18.1%)	21 (35%)	
COVID-19 misconceptions, *M* (*SD*)	0.95 (1.16)	0.84 (1.06)	1.32 (1.36)	0.02^*^
Parent COVID-19 safety measures, *M* (*SD*)	4.37 (1.86)	4.46 (1.78)	4.10 (2.07)	0.24
General vaccine mistrust, *M* (*SD*)	3.32 (1.26)	3.34 (1.26)	3.28 (1.28)	0.77
COVID-19 collectivist attitudes, *M* (*SD*)	4.08 (1.52)	4.20 (1.52)	3.72 (1.50)	0.03^*^
COVID-19 individualist attitudes, *M* (*SD*)	3.24 (1.78)	3.14 (1.76)	3.53 (1.83)	0.14

*Statistical tests: Independent t-tests for parent age, COVID-19 misconceptions, parent COVID-19 safety measures, general vaccine mistrust, COVID-19 collectivist attitudes, and COVID-19 individualist attitudes; Chi-square tests of independence for all other variables*.

a *“No” and “I don't know” combined in Chi-square analyses*.

* *Indicates significance, p < 0.05*.

## Results

**Figure 1:**
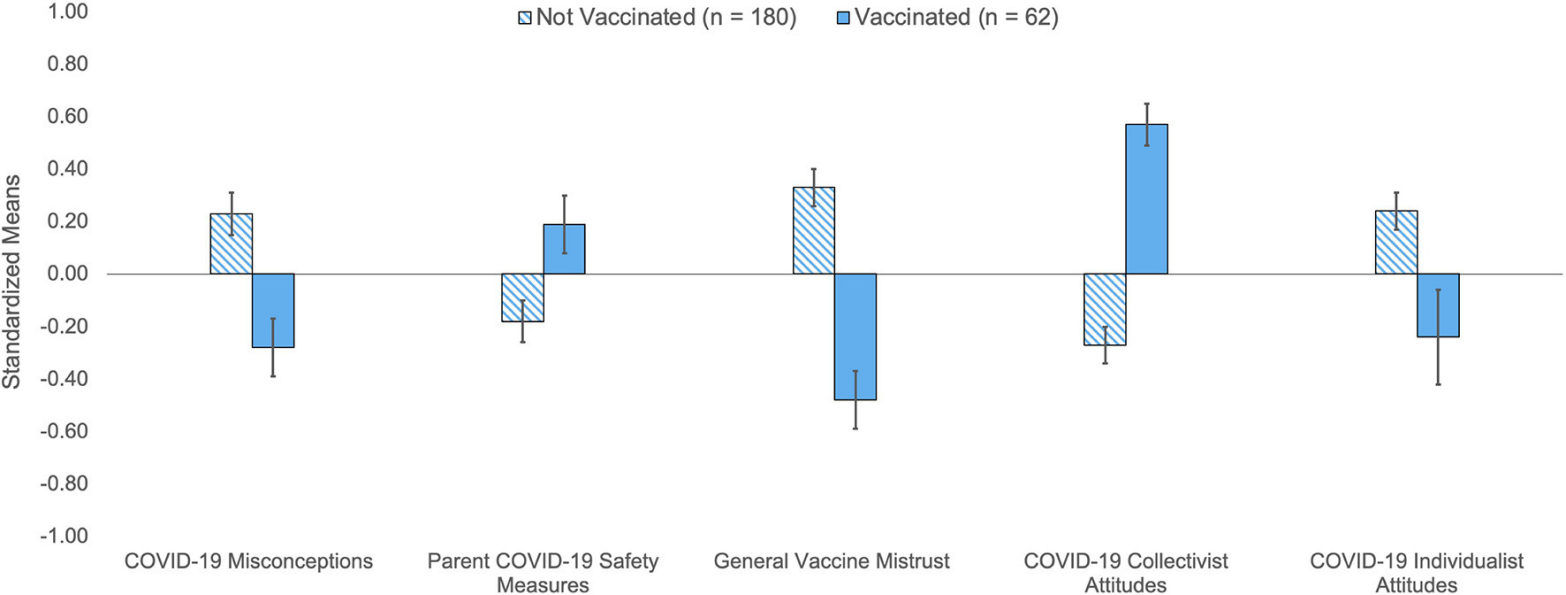
Differences in standardized scale means for COVID-19 attitudes and beliefs by vaccination status among adolescents ages 12–17. Standardized means based on z-scores for COVID-19 attitudes and vaccine beliefs indicate that parents whose older adolescent child ages 12–17 years old was unvaccinated reported above average COVID-19 misconceptions, general vaccine mistrust, and COVID-19 individualist attitudes where parent COVID-19 safety measures and COVID-19 collectivist attitudes were below average. The opposite was true for parents whose child was vaccinated on all scales. Errors bars represent standard error means and 0 in the y-axis denotes the mean score. Independent *t*-tests indicated that both groups were significantly different from each other on all scales, *p* ≤ 0.001 to *p* = 0.02.

Only 25.6% (*n* = 62) of respondents (*N* = 242) indicated their 12–17 year old adolescent had received the COVID-19 vaccine compared to 74.4% (*n* = 180) who indicated that their child was unvaccinated. As reported in Table 1, the odds of vaccination were higher for parents who were older, already vaccinated, required to be vaccinated by their employer, or living with other vaccinated adults. Across race/ethnicity, parents who had vaccinated their adolescent endorsed significantly fewer COVID-19 misconceptions, less general vaccine mistrust, and less COVID-19 individualist attitudes. These parents engaged in more COVID-19 safety measures and expressed greater COVID-19 collectivist attitudes. Figure 1 illustrates differences in standardized scale means between parents whose adolescent had or had not been vaccinated.

As reported in Table 1, non-Hispanic White parents reported reduced odds of vaccinating their adolescent compared to non-Hispanic Asian, non-Hispanic Black, and Hispanic parents. Further ANOVA and chi-square analyses revealed variable racial/ethnic differences in annual household income and financial security, vaccination status of the respondent and other adults living in the same household, and endorsement of COVID-19 misconceptions. No other differences were found among parent demographics or COVID-19 attitudes and beliefs across all four racial/ethnic groups. Race/ethnic group percentages and ANOVA and chi-square results for all variables are reported in the Supplementary Table 1 for this report.

To better understand factors underlying differences in adolescent uptake between non-Hispanic White parents and other racial/ethnic groups, we combined the responses of Asian, Black, and Hispanic parents and compared them to the responses of non-Hispanic White parents (see Table 2). Our data indicated that just 13.3% of White parents had vaccinated their adolescent compared to a significantly higher proportion (29.7%) of the other race/ethnicity parents. Non-Hispanic White parents also endorsed significantly more COVID-19 misconceptions and significantly lower COVID-19 collectivist attitudes. There were no other significant racial/ethnic differences among parent characteristics when comparing the combined Asian, Black, and Hispanic parent responses to non-Hispanic White parent responses.

A multivariable binomial logistic regression that included parent age, race/ethnicity, parent vaccine status, COVID-19 misconceptions, general vaccine mistrust, COVID-19 safety measures, and collectivist and individualist attitudes explained 46% (Nagelkerke R^2^) of the variance in whether adolescents had been vaccinated against COVID-19 infection. Table 3 reports betas and standard errors, *p*-values, odds ratios, and 95% confidence intervals for factors included in the binomial logistic model. Parental age, vaccine status, and endorsement of COVID-19 collectivist attitudes independently increased the odds of vaccination among 12–17 year old children whereas identifying as non-Hispanic White and endorsing general vaccine mistrust independently decreased the odds of vaccination. Since parental vaccine status was identified as a prominent explanatory determinant of adolescent vaccine uptake, we conducted bivariate correlations examining the relationship between parental vaccine status and other explanatory variables. Results indicate that parental vaccine status was positively correlated with older parent age (*r* = 0.19), greater financial security (*r* = 0.20), having other vaccinated adults in the household (*r* = 0.37), reporting greater engagement in COVID-19 safety measures (*r* = 0.21), and COVID-19 collectivist attitudes (*r* = 0.41) while being negatively correlated with COVID-19 misconceptions (*r* = −0.25), general vaccine mistrust (*r* = −0.43), and COVID-19 individualist attitudes (*r* = −0.29). Correlations between all explanatory variables are provided in the Supplementary Table 2 for this report.

**Table 3:** Adjusted binomial logistic regressions predicting vaccination status of adolescent child ages 12–17.

**Variable**	***B* (*SE*)**	***P*-value**	**OR (95% CI)**
Parent age	0.07 (0.02)	0.01*	1.07 (1.02, 1.12)
Race/ethnicity (Hispanic and Non-Hispanic Asian and Black compared to Non-Hispanic White)	−1.20 (0.50)	0.02*	0.30 (0.11, 0.80)
Parent vaccine status	1.98 (0.50)	<0.001*	7.22 (2.74, 19.05)
Parent COVID-19 safety measures	−0.05 (0.12)	0.68	0.95 (0.76, 1.19)
COVID-19 misconceptions	−0.23 (0.22)	0.32	0.79 (0.50, 1.26)
General vaccine mistrust	−0.48 (0.24)	0.05*	0.62 (0.39, 1.00)
COVID-19 individualist attitudes	0.25 (0.16)	0.11	1.29 (0.95, 1.76)
COVID-19 collectivist attitudes	0.41 (0.17)	0.02*	1.51 (1.08, 2.11)
Parent's employer requires vaccination	−0.17 (0.44)	0.70	0.84 (0.35, 2.00)
Other adults in their household are vaccinated	0.08 (0.19)	0.66	1.09 (0.75, 1.58)

OR, odds ratio; 95% CI, 95% confidence interval; Nagelkerke R^2^ = 0.46. The * symbol indicates significance p < 0.05.

## Discussion

Despite progress in COVID-19 vaccination rates among 12–17 year-olds since FDA emergency authorization in May 2021, to date, 9.9 million or nearly half of U.S. adolescents remain unvaccinated (5–7) in comparison to 61–85% of U.S. adults depending on age group (33). As such, concerns about parental pediatric vaccine refusal are growing. In the current brief report, just 25.6% of parents from diverse racial/ethnic backgrounds in the U.S. reported that their 12–17 year-old had been vaccinated against COVID-19 infection. This percentage is markedly lower than the 51% total U.S. adolescent vaccination rate (5). However, the demographic characteristics of our sample are consistent with factors associated with parental vaccine hesitancy: 29.8% did not attend college, 40.1% reported < $20,000 in household income, 24.8% endorsed the item “I cannot make ends meet” and 64% lived in the Midwest and South (6, 8–13, 34–36). This study contributes to the growing body of literature on pediatric COVID-19 vaccinations by highlighting characteristics and attitudes that independently and conjointly influence parental vaccine hesitancy and identifying how these determinants and decisions to vaccinate vary across U.S. racial/ethnic groups.

Among our sample, bivariate analyses indicated that race/ethnicity, parental age, vaccine status, employer requirements for vaccination, and vaccination among other adults in the household were significant social determinants of vaccine uptake among 12–17 years old. Among parents who themselves were vaccinated, the unadjusted odds of vaccine uptake for their older child were 14 times higher than unvaccinated parents. Our findings are consistent with previous research reporting that vaccinated parents are more likely than unvaccinated parents to accept the vaccine for their children (6, 8–10, 12, 16, 17, 19, 21, 22). Further, our data point to the influence of one's larger social context on vaccine acceptance. The unadjusted odds of vaccine uptake were twice as high for parents whose employer required vaccination and 7 times higher for parents living with other vaccinated adults. Taken together, our results highlight the importance of considering the ways in which social context normalizes COVID-19 vaccination among parents. Further research is needed to better understand what factors motivate parent COVID-19 vaccine uptake and how these contexts impact parental pediatric COVID-19 vaccine acceptance and hesitancy in general in addition to assessing the extent to which racial/ethnic group identification influences these relationships.

Bivariate analyses also indicated that COVID-19 attitudes and beliefs among parents were significant determinants of adolescent vaccine uptake. Among our sample, parental COVID-19 misconceptions, general vaccine mistrust, and COVID-19 individualist attitudes were found to decrease the odds of vaccine uptake whereas COVID-19 safety measures and collectivist attitudes improved the odds of vaccine uptake. Previous research has found that parents' misconceptions about COVID-19 transmission, symptoms, and severity, and their general attitudes about pediatric vaccine safety are significant barriers to vaccine acceptance (6, 8–11, 13–21). Consequently, future research must identify public health measures that are effective in reducing vaccine misconceptions and mistrust while also being sensitive to differences in the influence of these concerns across different racial/ethnic groups of parents.

Our data are consistent with previous research indicating COVID-19 safety measures such as wearing a mask in public, social distancing, and frequent handwashing are associated with COVID-19 vaccine acceptability among parents (22). While recent data suggests that parents in the U.S. may value personal choice over collective responsibility (6), our data indicate that individualistic and collectivist attitudes play competing roles in parental acceptance of COVID-19 vaccines for children. Understanding the relationships between pediatric vaccine acceptance and the inter-relationships among parental COVID-19 safety behaviors and collectivist and individualist community attitudes is a necessary step for improved public health messaging.

Among our sample, very few racial/ethnic differences were found in parent demographics and COVID-19 vaccine behaviors, attitudes, and beliefs. What did emerge was the finding that in comparison to non-Hispanic Asian, non-Hispanic Black, and Hispanic parents, non-Hispanic White parents were more likely to report that their adolescent had not been vaccinated, and further, were more likely to endorse COVID-19 misconceptions and reject COVID-19 collectivist attitudes. These results are in contrast to reports conducted early in the pandemic which found greater vaccine hesitancy among Hispanic and non-Hispanic Black parents (4, 9–13). However, more recent data suggest a shift in attitudes among adults of color in the U.S. In particular, these findings indicated that Black adults have seemingly “overcome” vaccine hesitancy at a faster pace than White adults over the course of the pandemic (37). Longitudinal data demonstrated that beliefs that COVID-19 vaccines are safe, effective, and necessary to protect oneself and one's community was predictive of personal intentions to receive the COVID-19 vaccinate with Black adults experiencing a faster shift in attitudes than White adults. Findings of the current and past research underscore an urgent need to consider racial/ethnic differences in COVID-19 vaccine concerns and attitudes among parents in order to develop effective public health communication strategies.

### Limitations

This brief report is not without limitations. Our findings are based on cross-sectional data which cannot assess causal effects over time of these determinants on parents' pediatric vaccine decisions. Further, participant recruitment and participation were conducted entirely online through a survey panel aggregator, and consequently, participation was limited to individuals who have access to the internet on web-enabled devices and also who have signed up to complete surveys for compensation. Additionally, we observed among this sample that no participant reported COVID-19 infections among family members living in their household: 78% indicated no infections at all and 22% indicated that they don't know if any family member were ever infected. There are a few possible reasons for this. First, about half of the current sample reported being vaccinated and living with family members who are vaccinated. For some individuals, it is likely that their family members have never been infected with COVID-19; and for others, family members could have been infected and not have known due to increased odds of asymptomatic symptomatology among vaccinated individuals. Further, persistent low testing rates and inaccessibility of testing in the U.S. can also mean that participants and their family members were simply not being tested for COVID-19 infection. Although we do not have data on family member testing behaviors, we see glimpses of inadequate testing among our sample. Among parents, although a little more than 50% reported never being infected and receiving negative COVID-19 test results to confirm, 37% reported never having been infected but never being tested for COVID-19 infection and the few parents who did report previous infections (*n* = 16, 7%) indicated that they had never been tested as well. As a brief report drawn from data available from a larger study, our sample was limited in that we were only able to provide data from parents among our larger sample who reported having both a child between the ages of 5–11 and 12–17, and we did not have demographic data including age or gender of the 12–17 year old child, although these characteristics have not been reported as significant in other studies (10, 12). Finally, although our study was nationally representative, we did not assess rural, suburban, or urban differences which may also be related to vaccine hesitancy or acceptance.

## Conclusions

Stemming the tide of the ongoing and ever-evolving COVID-19 pandemic depends on sufficient vaccination rates among all age groups. For children, vaccine uptake is contingent on hesitancy or acceptance among their parents. This brief report identifies parental sociodemographic differences, behaviors, and attitudes that have unique and inter-related effects on COVID-19 vaccine uptake among 12–17 year-olds. Our findings indicate that among these factors, increasing parental vaccine uptake, promoting COVID-19 vaccine collectivist attitudes, leveraging individualist attitudes, and alleviating general vaccine mistrust within the context of distinct racial/ethnic communities will be instrumental to public health efforts to improve vaccination uptake among adolescents. Our findings also suggest that future research can benefit from purposive sampling that includes sufficient numbers of racial/ethnic groups characteristic of the U.S. demographic mosaic. Continuing to assess racial/ethnic differences is necessary if we are to overcome vaccine refusal currently stunting progress in vaccination among pediatric populations. As such, public health efforts must consider the unique attitudes, beliefs, and concerns among racial groups and target differential sources of misinformation, vaccine disinterest, and vaccine mistrust most likely to be antecedents of vaccine hesitancy among distinct racial/ethnic parental groups. These efforts should utilize culturally relevant messaging campaigns that emphasize both community and personal protection as a larger aspect of ongoing public health efforts to curb COVID-19 infection rates. Future national and local government efforts must also be directed at regaining public trust in public health messaging and improving vaccine science literacy. Overall, these efforts will require an understanding of the unique barriers and facilitators contributing to parental vaccine hesitancy that can inform the effective population tailored public health messaging and interventions needed to improve pediatric COVID-19 vaccine uptake if we are to return to pre-pandemic normalcy.

## Data Availability Statement

The raw data supporting the conclusions of this article will be made available by the authors and for data files please contact AG, agray11@fordham.edu.

## Ethics Statement

This study involving human participants was reviewed and approved by the Fordham University Institutional Review Board. The participants indicated informed consent to participate in this study.

## Author Contributions

AG and CF: conceptualization, methodology, original draft preparation, writing-review, and editing. AG: data visualization and formal analysis. All authors have read and agreed to the published version of the manuscript.

## Conflict of Interest

The authors declare that the research was conducted in the absence of any commercial or financial relationships that could be construed as a potential conflict of interest.

## Publisher's Note

All claims expressed in this article are solely those of the authors and do not necessarily represent those of their affiliated organizations, or those of the publisher, the editors and the reviewers. Any product that may be evaluated in this article, or claim that may be made by its manufacturer, is not guaranteed or endorsed by the publisher.
