# Supplementary material for: Determinants of COVID-19 Vaccine Uptake in Adolescents 12–17 Years Old: Examining Pediatric Vaccine Hesitancy Among Racially Diverse Parents in the United States
Source: Front Public Health. 2022 Mar 22;10:844310. doi: 10.3389/fpubh.2022.844310 (PMC8980347; doi:10.3389/fpubh.2022.844310)
Supplement: Supplementary file 1 [file Data_Sheet_1.docx]

Gray, A., & Fisher, C. B (2022)

Determinants of COVID-19 vaccine uptake in adolescents 12 – 17 years old: Examining pediatric vaccine hesitancy among racially diverse parents in the United States

Frontiers in Public Health: Children and Health Section

***Supplemental Materials***

**Table SM1.** Frequencies/percentages and means/standard deviations for racial/ethnic group differences in adolescent vaccination status and parent characteristics

|  | **Total Sample**  (*N* = 242) | **Non-Hispanic Asian Parents**  (*N* = 48) | **Non-Hispanic Black Parents** (*N* = 63) | **Hispanic Parents**  (*N* = 71) | **Non-Hispanic White Parents** (*N* = 60) | ***p*-value** |
| --- | --- | --- | --- | --- | --- | --- |
|  | ***N* (%)** | ***N* (%)** | ***N* (%)** | ***N* (%)** | ***N* (%)** |  |
| **Vaccination status of adolescent (ages 12 to 17)** |  |  |  |  |  | .08 |
| No | 180 (74.4%) | 34 (70.8%) | 46 (73%) | 48 (67.6%) | 52 (86.7%) |  |
| Yes | 62 (25.6%) | 14 (29.2%) | 17 (27%) | 23 (32.4%) | 8 (13.3%) |  |
| **Parent age,** *M* (*SD*) | 35.67 (7.74) | 35.35 (5.81) | 35.75 (7.57) | 34.82 (7.57) | 36.95 (9.32) | .45 |
| **Education** |  |  |  |  |  | .71 |
| Did not attend college | 72 (29.8%) | 12 (25%) | 22 (34.9%) | 21 (29.6%) | 17 (28.3%) |  |
| Some college or higher | 170 (70.2%) | 36 (75%) | 41 (65.1%) | 50 (70.4%) | 43 (71.7%) |  |
| **Annual household income** |  |  |  |  |  | < .001* |
| Less than $20,000 | 97 (40.1%) | 16 (33.3%) | 30 (47.6%) | 23 (32.4%) | 28 (46.7%) |  |
| Between $20,000 and $50,999 | 111 (45.9%) | 22 (45.8%) | 29 (46%) | 36 (50.7%) | 24 (40%) |  |
| Between $51,000 and $79,999 | 22 (9.1%) | 1 (2.1%) | 2 (3.2%) | 12 (16.9%) | 7 (11.7%) |  |
| Preferred not to answer | 12 (5%) | 9 (18.8%) | 2 (3.2%) | 0% | 1 (1.7%) |  |
| **Financial security** |  |  |  |  |  | .02* |
| Cannot make ends meet | 60 (24.8%) | 6 (12.5%) | 24 (38.1%) | 15 (21.1%) | 15 (25%) |  |
| Have just enough or comfortable | 182 (75.2%) | 42 (87.5%) | 39 (61.9%) | 56 (78.9%) | 45 (75%) |  |
| **Region of residence** |  |  |  |  |  | .31 |
| Northeast | 34 (14%) | 2 (4.2%) | 8 (12.7%) | 10 (14.1%) | 14 (23.3%) |  |
| Midwest | 86 (35.5%) | 20 (41.7%) | 22 (24.9%) | 25 (35.2%) | 19 (31.7%) | . |
| South | 70 (28.9%) | 14 (29.2%) | 22 (34.9%) | 18 (25.4%) | 16 (26.7%) |  |
| West | 52 (21.5%) | 12 (25%) | 11 (17.5%) | 18 (25.4%) | 11 (18.3%) |  |
| **Parent vaccine status** |  |  |  |  |  | .01* |
| No | 123 (50.8%) | 17 (35.4%) | 41 (65.1%) | 32 (45.1%) | 33 (55%) |  |
| Yes | 119 (49.2%) | 31 (64.6%) | 22 (34.9%) | 39 (54.9%) | 27 (45%) |  |
| **Parent’s employer requires vaccination^a^** |  |  |  |  |  | .09 |
| No | 194 (80.2%) | 36 (75%) | 51 (81%) | 53 (74.6%) | 54 (90%) |  |
| Yes | 42 (17.4%) | 11 (22.9%) | 8 (12.7%) | 17 (23.9%) | 6 (10%) |  |
| I don’t know | 6 (2.5%) | 1 (2.1%) | 4 (6.3%) | 1 (1.4%) | 0% |  |
| **Other adults in their household are vaccinated^a^** |  |  |  |  |  | .001* |
| No | 136 (52.1%) | 15 (31.3%) | 45 (71.4%) | 33 (46.5%) | 33 (55%) |  |
| Yes | 114 (47.1%) | 32 (66.7%) | 18 (28.6%) | 37 (52.1%) | 27 (45%) |  |
| I don't know | 2 (.8%) | 1 (2.1%) | 0% | 1 (1.4%) | 0% |  |
| **Family members in their household ever had COVID-19** |  |  |  |  |  | –– |
| No | 188 (77.7%) | 40 (83.3%) | 52 (82.5%) | 57 (80.3%) | 39 (65%) |  |
| Yes | 0% | 0% | 0% | 0% | 0% |  |
| I don't know | 54 (22.3%) | 8 (16.7%) | 11 (17.5%) | 14 (19.7%) | 21 (35%) |  |
| **COVID-19 misconceptions**, *M* (*SD*) | .95 (1.16) | 1.00 (1.20) | .73 (1.07) | .83 (.96) | 1.32 (1.36) | .03* |
| **Parent COVID-19 safety measures,** *M* (*SD*) | 4.37 (1.86) | 4.35 (1.78) | 4.90 (1.60) | 4.13 (1.87) | 4.10 (2.07) | .05 |
| **General vaccine mistrust,** *M* (*SD*) | 3.32 (1.26) | 3.08 (1.29) | 3.48 (1.10) | 3.37 (1.35) | 3.28 (1.28) | .41 |
| **COVID-19 collectivist attitudes,** *M* (*SD*) | 4.08 (1.52) | 4.27 (1.43) | 3.98 (1.57) | 4.35 (1.52) | 3.72 (1.50) | .08 |
| **COVID-19 individualist attitudes,** *M* (*SD*) | 3.24 (1.78) | 3.13 (1.71) | 3.15 (1.70) | 3.13 (1.86) | 3.53 (1.83) | .52 |

*Note.* Statistical tests: ANOVAs for parent age, COVID-19 misconceptions, parent COVID-19 safety measures, general vaccine mistrust, COVID-19 collectivist attitudes, and COVID-19 individualist attitudes; Chi-square tests of independence for all other variables.

^a^“No” and “I don’t know” combined in Chi-square analyses
*indicates significance, *p* < .05

**Table SM2.** Bivariate correlations among potential determinants of vaccination status among adolescent children ages 12–17

|  |  | 1 | 2 | 3 | 4 | 5 | 6 | 7 | 8 | 9 | 10 | 11 | 12 | 13 |
| --- | --- | --- | --- | --- | --- | --- | --- | --- | --- | --- | --- | --- | --- | --- |
| 1. | Parental vaccine status (no compared to yes) | — |  |  |  |  |  |  |  |  |  |  |  |  |
| 2. | Parent age | .15** | — |  |  |  |  |  |  |  |  |  |  |  |
| 3. | Race/ethnicity (Hispanic and Non-Hispanic Asian and Black compared to Non-Hispanic White) | -.10* | .12* | — |  |  |  |  |  |  |  |  |  |  |
| 4. | College education | .15** | .03 | -.03 | — |  |  |  |  |  |  |  |  |  |
| 5. | Financial security | .16** | -.08 | .01 | .18*** | — |  |  |  |  |  |  |  |  |
| 6. | Region of residence (Midwest, South, and Northeast compared to Western U.S.) | .02 | .05 | -.03 | -.11* | -.004 | — |  |  |  |  |  |  |  |
| 7. | Parent employer requires vaccination (no compared to yes) | .09 | -.003 | -.09 | -.05 | -.09 | .06 | — |  |  |  |  |  |  |
| 8. | Other adults in their household are vaccinated (no compared to yes) | .26*** | .02 | .01 | .05 | -.03 | .06 | .13* | — |  |  |  |  |  |
| 9. | COVID-19 misconceptions | -.29*** | -.05 | .13* | -.06 | -.01 | -.01 | -.01 | -.03 | — |  |  |  |  |
| 10. | Parent COVID-19 safety measures | .25*** | .14** | -.16*** | .05 | -.07 | .06 | .02 | .04 | -.36*** | — |  |  |  |
| 11. | General vaccine mistrust | -.44*** | -.10* | .002 | -.14** | -.10* | .04 | .02 | -.09 | .43*** | -.20*** | — |  |  |
| 12. | COVID-19 collectivist attitudes | .49*** | .03 | -.16*** | .21** | .11* | -.05 | -.002 | .11* | -.41*** | .22*** | -.53** | — |  |
| 13. | COVID-19 individualist attitudes | -.37*** | -.09 | .06 | -.08 | -.05 | .01 | -.003 | -.10* | .41*** | -.20*** | .59** | -.42*** | — |

**p* < .05 ***p* ≤ .01 ****p* ≤ .001
